# Long-Term Recurrence and the Safety of Mesh Use After Emergency Ventral Hernia Repair

**DOI:** 10.1001/jamanetworkopen.2025.44303

**Published:** 2025-11-18

**Authors:** Erin E. Isenberg, Brian T. Fry, Joshua Sinamo, Ryan A. Howard, Anne P. Ehlers, Jenny M. Shao, Raymond A. Jean, Dana A. Telem

**Affiliations:** 1 Department of Surgery, The University of Texas at Southwestern Medical Center, Dallas; 2 National Clinician Scholars Program, Institute for Healthcare Policy and Innovation, University of Michigan, Ann Arbor; 3 Department of Surgery, University of Michigan Medical School, Ann Arbor

## Abstract

**Importance:**

Wide variation exists in how surgeons approach the repair of emergent presentation of ventral hernias, with many opting to avoid mesh in the acute setting. The long-term recurrence rates after emergent hernia repair and the effects of mesh on safety and recurrence remain poorly characterized.

**Objective:**

To evaluate the long-term reoperation rate for recurrence and safety of mesh use after emergent ventral hernia repair.

**Design, Setting, and Participants:**

This retrospective cross-sectional study of adults aged 18 years or older who underwent emergent inpatient ventral hernia repair used 100% Medicare administrative claims data from January 1, 2011, to December 31, 2021. Patients were followed up through death or termination of Medicare insurance. The data analysis was conducted between April and December 2024.

**Exposures:**

Open hernia repair with or without mesh use and with or without enterectomy.

**Main Outcomes and Measures:**

The primary outcome was the reoperation rate for recurrence after open emergent hernia repair stratified by mesh use. The secondary outcome was mesh explantation rates for emergent open repairs performed with and without enterectomy. The time-to-event analysis used Royston-Parmar models.

**Results:**

A total of 122 651 adult Medicare beneficiaries (mean [SD] age, 71.4 [21.0] years; 71 463 females [58.3%]) underwent emergent ventral hernia repair from 2011 to 2021. The median time to the follow-up event after the index hernia surgery was 3.2 years (IQR, 1.1-6.0 years). The 10-year reoperation rate for recurrence after emergency ventral hernia repair was 16.3% (95% CI, 15.9%-16.6%). Among emergent open repairs, 30 922 (30.0%) were repaired with mesh. Of those undergoing emergent open repairs with enterectomy, 2055 patients (18.2%) received mesh. Overall, patients repaired with mesh had significantly lower 10-year recurrence rates than those repaired without mesh (13.0% [95% CI, 13.0%-13.8%] vs 18.9% [18.4%-18.9%]; hazard ratio [HR], 0.66 [95% CI, 0.63-0.69];* P* < .001). Mesh explantation rates up to 10 years after surgery were not significantly different between emergent open repairs that did and did not require enterectomy (3.8% [95% CI, 2.8%-4.8%] vs 3.2% [95% CI, 2.9%-3.4%]; HR, 1.20 [95% CI, 0.91-1.59]).

**Conclusions and Relevance:**

In this cross-sectional study of patients undergoing emergent open hernia repairs, mesh use was associated with decreased risk of recurrence and was not associated with increased risk of mesh explantation in the setting of enterectomy. This study suggests that surgeons may consider more consistent use of mesh in the emergent setting to possibly improve long-term outcomes.

## Key Points

**Question:**

How is mesh use associated with the long-term reoperation rate for recurrence after emergency open ventral hernia repair?

**Findings:**

In this cross-sectional study of 122 651 adults, the 10-year reoperation rate for recurrence after emergency ventral hernia repair was 16.3%. Among emergent open repairs, those repaired with mesh had significantly lower 10-year recurrence rates than those repaired without mesh (13.0% vs 18.9%).

**Meaning:**

The findings of this study suggest that mesh use was associated with lower long-term reoperation for recurrence after emergent open ventral hernia repair, which may improve long-term outcomes in this setting.

## Teaser

This cross-sectional study assesses the long-term reoperation rate for recurrence after emergent ventral hernia repair with or without mesh use.

## Introduction

Emergency ventral hernia repair is known to be associated with higher morbidity and mortality when compared with repairs performed in an elective fashion.^1^ Emergent repairs are often done in nonideal settings, such as in patients who are acutely ill or have bowel compromise, without adequate preoperative planning or comorbidity optimization, or with more limited operative resources. Thus, management of emergent hernia repair is often subject to different approaches than would be undertaken in the elective setting and may entail avoiding upfront definitive repairs.^2^ Ultimately, these factors contribute to substantial variability in practice patterns or a lack of clarity around how to best manage these patients and may potentiate worse outcomes in this population.

Knowledge of the incidence of and outcomes after emergency ventral hernia repair remains limited for several important reasons. First, there is lack of contemporary insight into population-level long-term recurrence rates after emergency ventral hernia repair, as most studies are limited to elective cases, small cohorts, or short-term outcomes.^3,4,5,6,7^ Additionally, while mesh use is known to be safe and reduce recurrence in elective hernia repairs, there are limited data on the long-term safety and efficacy of mesh use in emergent cases, which are more likely to be clean-contaminated or contaminated cases.^8^ This lack of high-quality data to guide decision-making contributes to an inability to optimize approaches and outcomes in this high-risk group.

This study sought to characterize the association of mesh use with the long-term reoperation rate for recurrence after emergency ventral hernia repair in a national cohort of Medicare beneficiaries. The use of Medicare enabled a population-level analysis and the ability to measure long-term operative hernia recurrence and mesh explantation rates due to Medicare’s near-universal continuous enrollment. A better understanding of the incidence of and outcomes after emergency ventral hernia repair has the potential to critically inform the continued evolution in the management of ventral hernia.

## Methods

### Data Source and Patient Population

In this cross-sectional study, we used data from the 100% capture Medicare Provider Analysis and Review parts A and B files to identify initial (index) operations for persons aged 18 or older who underwent emergent inpatient ventral hernia repair from January 1, 2011, through December 31, 2021. Patients were followed up until death or disenrollment from Medicare. Emergent procedures were determined by an urgent or emergent admission status as defined by the Claim Inpatient Admission Type Code, validated in prior surgical studies using Medicare claims.^9,10^ This study was exempt from regulation by the University of Michigan institutional review board, and informed consent was waived because of the use of deidentified data per the institutional review board policies. This study followed the Reporting of Studies Conducted Using Observational Routinely Collected Data (RECORD) statement.

Ventral hernia included all anterior abdominal wall hernias coded in claims as ventral, incisional, umbilical, or epigastric hernia repair. Patients were identified initially using the *International Classification of Diseases, Ninth Revision *(*ICD-9*) and the *International Statistical Classification of Diseases and Related Health Problems, Tenth Revision *(*ICD-10*) procedure codes, which were cross-referenced with the corresponding *ICD-9* and *ICD-10* diagnosis codes (eTable 1 in Supplement 1).

Patients were excluded from the index cohort if they had a prior hernia repair in at least the 2 years leading up to the initial operation or if the repair was associated with *Current Procedural Terminology* (*CPT*) codes for repair of a recurrent ventral hernia (49565, 49566, 49656, and 49657). All subsequent admissions for a ventral hernia operation after the index repair were excluded from the index cohort. Patients were also excluded if they were not enrolled in a Medicare Fee-For-Service plan (due to lack of accurate follow-up) or if they lacked a concurrent *CPT* code for hernia repair (eTable 1 in Supplement 1). A flow diagram with detailed stepwise patient inclusion and exclusion criteria is presented in eFigure 1 in Supplement 1.

### Outcome Measures and Explanatory Variables

We first characterized overall long-term reoperation rates for recurrence for emergent hernia repairs and reoperation rates for recurrence for emergent open umbilical and incisional-ventral hernia repairs. Operative recurrence was used instead of clinical or radiographic recurrence, which cannot be identified in Medicare claims data alone. Operative recurrence up to 10 years after initial surgery was identified by a subsequent hernia repair using the same *CPT* and *ICD-9* and *ICD-10* codes used to identify the initial hernia repair and/or the presence of specific hernia recurrence *CPT* codes (49565, 49566, 49656, and 49657), consistent with prior work.^7,11^

The primary outcome was the reoperation rate for recurrence after open emergent hernia repair stratified by mesh use. Because mesh use is presumed for both laparoscopic and robotic-assisted hernia repairs (and not reimbursed if coded separately), we restricted this portion of our analysis to only ventral hernias repaired with an open approach. The emergent repairs performed in an open fashion were stratified by mesh use (*CPT* code 49568), and the subsequent reoperation rate for recurrence was identified the same manner as above.

Our secondary outcome was the incidence of mesh explantation with and without enterectomy in this same cohort (open, emergent hernia repairs). This is particularly relevant for emergent hernia repairs, in which there is a higher likelihood of the Centers for Disease Control and Prevention class III (contaminated) or IV (dirty-infected) wounds due to bowel necrosis with enteric spillage or frank perforation, and evidence for the use of mesh is limited.^6^ *CPT* codes 11008 and 49402 were used to identify mesh explantation up to 5 years after emergent open ventral hernia repairs. Mesh explantation rates were stratified by the concurrent need for bowel resection (*CPT* code 44120) to identify any differences in cases likely to be identified as clean and clean-contaminated vs contaminated and dirty-infected.

Explanatory variables included in our models were consistent with prior published work on hernia and included patient age, sex, race and ethnicity, Elixhauser comorbidities, mesh use, the use of myofascial release, and hernia subtype (incisional-ventral or umbilical).^11,12^ The race and ethnicity data were collected from Medicare claims data. Categories included American Indian or Alaska Native, Asian, Black, Hispanic, White, other (no subcategories available), and unknown, and only 1 category could be self-selected. Race and ethnicity were included because they have been found to be associated with the presentation, management, and outcomes of hernias and hernia repair. Additional details on race and ethnicity identification are provided in the eMethods in Supplement 1. We also included an operative approach (open, laparoscopic, or robotic-assisted) as a covariate for our overall operative recurrence model. Age was treated as a continuous variable. All other variables were treated as categorical.

### Statistical Analysis

Data analysis was conducted between April and December 2024. Due to the binary, nonlinear outcome of operative hernia recurrence, we performed a time-to-event analysis using Royston-Parmar models. The models were constructed to calculate the cumulative incidences of operative hernia recurrence and mesh explantation using the standardized (or marginal) survival curves while adjusting for the following covariates: age, sex, race and ethnicity, comorbidities, hernia subtype, myofascial flap use, and mesh use. All modeling accounted for clustering at the hospital level. Patients were censored if they died, disenrolled from Medicare, or reached the end of the study period. We initiated the analyses using the Cox proportional hazards regression models and found some variables to fail the Cox proportional hazards assumptions tests using Schoenfeld residuals. Therefore, we opted to use Royston-Parmar models and allocated time-varying coefficients for the variables that previously failed the Cox proportional hazards assumptions tests including sex, diabetes with chronic complications, kidney failure, obesity, weight loss, and chronic blood loss anemia.^13^

All analyses were performed using Stata, version 18 (StataCorp LLC). Tests were 2-sided, and significance was set at *P* < .05.

## Results

### Patient Characteristics and Incidence of Hernia Repair

A total of 122 651 patients underwent emergent hernia repair during the study period (Table 1). The mean (SD) age of the cohort was 71.4 (12.0) years; 71 463 (58.3%) of the patients were female, and 51 188 (41.7%) were male. In terms of race and ethnicity, 899 (0.7%) were American Indian or Alaska Native, 884 (0.7%) were Asian, 14 261 (11.6%) were Black, 3123 (2.5%) were Hispanic, 100 832 (82.2%) were White, 1496 (1.2%) were of other race and ethnicity, and 1156 (0.9%) were unknown.

**Table 1.** Patient Demographics for Emergent Ventral Hernia Repairs From 2011 to 2021

Characteristic	Patients, No. (%) (N = 122 651)
Age, mean (SD), y	71.4 (12.0)
Sex	
Female	71 463 (58.3)
Male	51 188 (41.7)
Race and ethnicity	
American Indian or Alaska Native	899 (0.7)
Asian	884 (0.7)
Black	14 261 (11.6)
Hispanic	3123 (2.5)
White	100 832 (82.2)
Other^a^	1496 (1.2)
Unknown	1156 (0.9)
Elixhauser comorbidities^b^	
Chronic pulmonary disease	31 299 (25.5)
Coagulopathy	7531 (6.1)
Congestive heart failure	20 880 (17.0)
Deficiency anemias	23 626 (19.3)
Depression	14 254 (11.6)
Diabetes	27 465 (22.4)
Diabetes with chronic complications	13 600 (11.1)
Fluid and electrolyte disorders	47 280 (38.5)
Hypertension	92 307 (75.3)
Hypothyroidism	21 816 (17.8)
Kidney failure	23 290 (19.0)
Liver disease	9517 (7.8)
Metastatic cancer	3615 (2.9)
Obesity	36 147 (29.5)
Other neurological disorders	8721 (7.1)
Peripheral vascular disease	10 829 (8.8)
Rheumatoid arthritis or collagen vascular disease	4417 (3.6)
Solid tumor without metastasis	5213 (4.3)
Valvular disease	8902 (7.3)
Weight loss	11 185 (9.1)
Hernia subtype	
Incisional-ventral	82 357 (67.1)
Umbilical	40 294 (32.9)
Mesh use^c^	50 574 (41.2)
Myofascial flap use	5191 (4.2)
Operative approach	
Open	102 999 (84.0)
Laparoscopic	17 149 (14.0)
Robotic	2503 (2.0)
Year	
2011	13 831 (11.3)
2012	13 192 (10.8)
2013	13 126 (10.7)
2014	11 953 (9.7)
2015	11 996 (9.8)
2016	11 444 (9.3)
2017	10 641 (8.7)
2018	10 422 (8.5)
2019	9980 (8.1)
2020	8265 (6.7)
2021	7801 (6.4)

^a^ No subcategories were available.

^b^ Only the top 20 Elixhauser comorbidities by overall prevalence are displayed in the table.

^c^ Includes any open procedure with a separate billing claim for mesh placement or any minimally invasive procedure (robotic, laparoscopic), as mesh placement is included in the billing claim for these cases.

The median time from admission to surgery was 1 day (IQR, 0-2 days). The median time to the follow-up event after the index hernia surgery was 3.2 years (IQR, 1.1-6.0 years). The incidence of emergent inpatient ventral hernia repairs decreased from 2011 to 2021, from 46 per 100 000 persons per year (13 831) to 28 per 100 000 persons per year (7801) (Table 1 and eFigure 2 in Supplement 1).

### Overall Reoperation Rate for Recurrence

**Figure 1.**
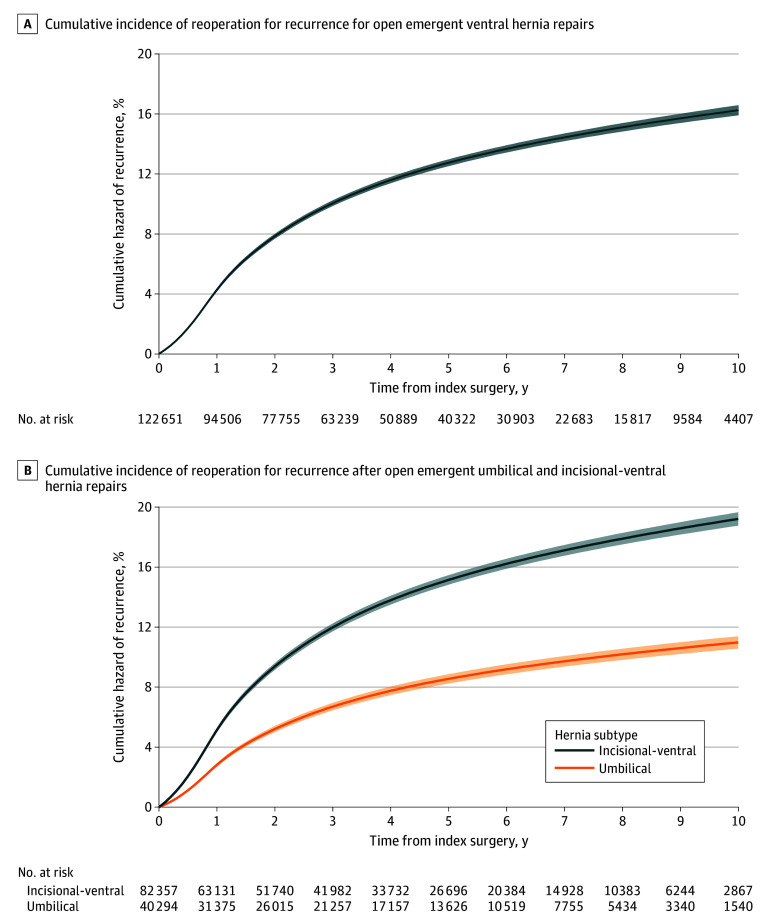
Cumulative Incidence and Umbilical and Incisional-Ventral Reoperation for Recurrence for Open Emergent Ventral Hernia Repairs From 2011 to 2021 The cumulative incidence of operative hernia recurrence was calculated using a Royston-Parmar flexible parametric survival model that adjusted for patient age, sex, race and ethnicity, Elixhauser comorbidities, mesh use, operative approach (A only), and hernia subtype (A only).

After adjustment for patient and operative covariates, the cumulative reoperation rate for recurrence for patients undergoing emergent repair was 10.0% (95% CI, 9.9%-10.1%) at 3 years, 12.7% (95% CI, 12.7%-12.8%) at 5 years, and 16.3% (95% CI, 15.9%-16.6%) at 10 years postoperatively (Figure 1A). For patients undergoing open, emergent operations, the cumulative reoperation rate for recurrence was 11.0% (95% CI, 10.6%-11.4%) for umbilical hernias and 19.2% (95% CI, 18.8%-19.7%) for incisional-ventral hernias at 10 years postoperatively (Figure 1B and eTable 2 in Supplement 1). The overall hazard ratio (HR) for umbilical vs incisional-ventral was 0.54 (95% CI, 0.52-0.57; *P* < .001).

### Mesh Use in Emergent Open Repairs

Characteristics for patients undergoing emergent open repair stratified by mesh use are presented in Table 2. A total of 30 992 (30.0%) of these repairs used mesh. Of the 11 305 patients undergoing enterectomy, only 2055 (18.2%) received mesh. In general, those undergoing repair with mesh were more likely to be younger and female and to have a lesser comorbidity burden except for higher rates of chronic pulmonary disease and obesity.

**Table 2.** Patient Demographics for Urgent and Emergent Ventral Hernia Repairs Performed via an Open Approach, by Mesh Use From 2011 to 2021

Characteristic	Patients, No. (%)			*P* value^b^
	All (N = 102 999)	Without mesh (n = 72 077)	With mesh (n = 30 922)^a^	
Age, mean (SD), y	71.6 (12.0)	71.9 (12.1)	70.9 (11.8)	<.001
Sex				
Female	59 411 (57.7)	38 991 (54.1)	20 420 (66.0)	<.001
Male	43 588 (42.3)	33 086 (45.9)	10 502 (34.0)	
Race and ethnicity				
American Indian or Alaska Native	732 (0.7)	507 (69.3)	225 (30.7)	.14
Asian	761 (0.7)	552 (72.5)	209 (27.5)	
Black	12 114 (11.8)	8550 (11.9)	3564 (11.5)	
Hispanic	2604 (2.5)	1816 (2.5)	788 (2.5)	
White	84 549 (82.1)	59 052 (81.9)	25 497 (82.5)	
Other^c^	1269 (1.2)	892 (1.9)	377 (1.2)	
Unknown	970 (0.9)	708 (1.0)	262 (0.8)	
Elixhauser comorbidities^d^				
Chronic pulmonary disease	26 344 (25.6)	17 739 (24.6)	8605 (27.8)	<.001
Coagulopathy	6679 (6.5)	5212 (7.2)	1467 (4.7)	<.001
Congestive heart failure	18 115 (17.6)	13 154 (18.2)	4961 (16.0)	<.001
Deficiency anemias	20 546 (19.9)	15 024 (20.8)	5522 (17.9)	<.001
Depression	11 862 (11.5)	7945 (11.0)	3917 (12.7)	<.001
Diabetes	23 218 (22.5)	15 807 (21.9)	7411 (24.0)	<.001
Diabetes with chronic complications	11 515 (11.2)	8384 (11.6)	3131 (10.1)	<.001
Fluid and electrolyte disorders	41 071 (39.9)	29 766 (41.3)	11 305 (36.6)	<.001
Hypertension	77 443 (75.2)	54 109 (75.1)	23 334 (75.5)	.19
Hypothyroidism	18 230 (17.7)	12 495 (17.3)	5735 (18.5)	<.001
Kidney failure	20 251 (19.7)	14 725 (20.4)	5526 (17.9)	<.001
Liver disease	8375 (8.1)	6810 (9.4)	1565 (5.1)	<.001
Obesity	29 827 (29.0)	19 297 (26.8)	10 530 (34.1)	<.001
Other neurological disorders	7440 (7.2)	5339 (7.4)	2101 (6.8)	<.001
Peripheral vascular disease	9525 (9.2)	7048 (9.8)	2477 (8.0)	<.001
Psychoses	3825 (3.7)	2601 (3.6)	1224 (4.0)	.007
Rheumatoid arthritis or collagen vascular disease	3710 (3.6)	2524 (3.5)	1186 (3.8)	.009
Solid tumor without metastasis	4562 (4.4)	3711 (5.1)	851 (2.8)	<.001
Valvular disease	7584 (7.4)	5527 (7.7)	2057 (6.7)	<.001
Weight loss	10 153 (9.9)	7520 (10.4)	2633 (8.5)	<.001
Hernia subtype				
Incisional-ventral	67 530 (65.6)	36 922 (51.2)	30 608 (99.0)	<.001
Umbilical	35 469 (34.4)	35 155 (48.8)	314 (1.0)	
Myofascial release	5007 (4.9)	1394 (1.9)	3613 (11.7)	<.001
Enterectomy	11 305 (11.0)	9250 (12.8)	2055 (6.6)	<.001
Year				
2011	12 018 (11.7)	8107 (11.2)	3911 (12.6)	<.001
2012	11 346 (11.0)	7705 (10.7)	3641 (11.8)	
2013	11 307 (11.0)	7671 (10.6)	3636 (11.8)	
2014	10 126 (9.8)	7056 (9.8)	3070 (9.9)	
2015	10 180 (9.9)	7155 (9.9)	3025 (9.8)	
2016	9657 (9.4)	6758 (9.4)	2899 (9.4)	
2017	8868 (8.6)	6244 (8.7)	2624 (8.5)	
2018	8575 (8.3)	6103 (8.5)	2472 (8.0)	
2019	8085 (7.8)	5844 (8.1)	2241 (7.2)	
2020	6676 (6.5)	4884 (6.8)	1792 (5.8)	
2021	6161 (6.0)	4550 (6.3)	1611 (5.2)	

^a^ Includes any open procedure with a separate billing claim for mesh placement or any minimally invasive procedure (robotic, laparoscopic), as mesh placement is included in the billing claim for these cases.

^b^ *P* values shown are for *t* test or Pearson χ^2^ test depending on variable type.

^c^ No subcategories were available.

^d^ Only the top 20 Elixhauser comorbidities by overall prevalence are displayed in the table.

**Figure 2.**
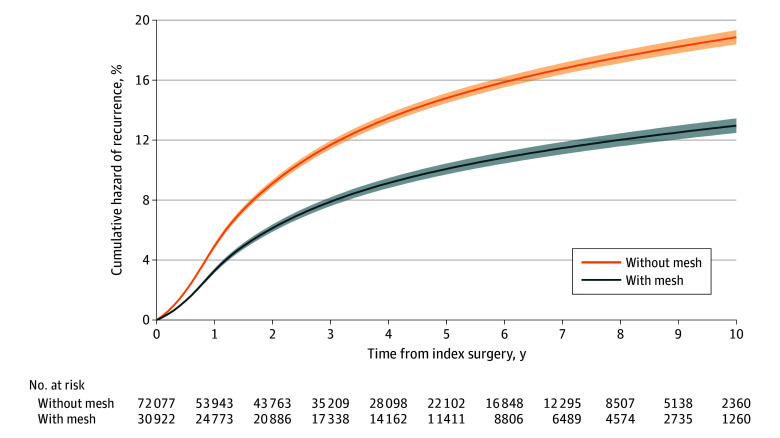
Cumulative Incidence of Reoperation for Recurrence for Open Emergent Ventral Hernia Repairs From 2011 to 2021, Stratified by Mesh Use The cumulative incidence of operative hernia recurrence was calculated using a Royston-Parmar flexible parametric survival model that adjusted for patient age, sex, race and ethnicity, Elixhauser comorbidities, and hernia subtype.

Of patients undergoing emergent open ventral hernia repair, those repaired with mesh had lower recurrence rates than those repaired without mesh (5 years with mesh: 10.1% [95% CI, 9.7%-10.4%] vs without mesh: 14.8% [95% CI, 14.5%-15.1%]; 10 years with mesh: 13.0% [95% CI, 13.0%-13.8%] vs without mesh: 18.9% [95% CI, 18.4%-18.9%]) (Figure 2 and eTable 2 in Supplement 1). The overall HR for mesh vs without mesh was 0.66 (95% CI, 0.63-0.69; *P* < .001).

**Figure 3.**
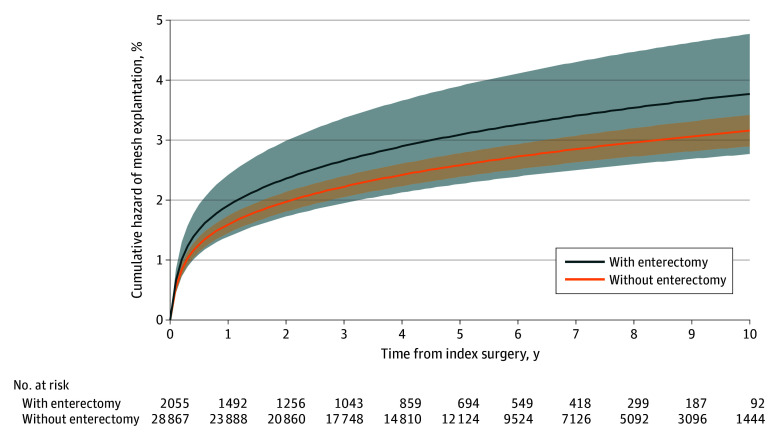
Cumulative Incidence of Mesh Explantation for Emergent Open Ventral Hernia Repairs From 2011 to 2021, Stratified by Concurrent Enterectomy The cumulative incidence of mesh explantation was calculated using a Royston-Parmar flexible parametric survival model that adjusted for patient age, sex, race and ethnicity, Elixhauser comorbidities, mesh use, and hernia subtype. The shaded areas indicate 95% CIs.

When further stratifying this cohort by those who underwent enterectomy, 5-year (3.2% [95% CI, 2.3%-4.0%] vs 2.6% [95% CI, 2.4%-2.8%]) and 10-year (3.8% [95% CI, 2.8%-4.8%] vs 3.2% [95% CI, 2.9%-3.4%]) mesh explantation rates were not significantly different for repairs that required a concurrent enterectomy vs those without enterectomy (Figure 3 and eTable 2 in Supplement 1). The overall HR for enterectomy vs no enterectomy was 1.20 (95% CI, 0.91-1.59; *P* = .21).

## Discussion

This cross-sectional study had several key findings. First, patients undergoing emergent hernia repair with mesh were significantly less likely to experience reoperation for recurrence than those repaired without mesh. Second, mesh explantation rates were not significantly different for these emergent cases when a concurrent enterectomy was performed. Lastly, overall long-term reoperation rates for recurrence for emergent hernia repairs were comparable with those reported for elective hernia repairs, indicating that they may be less likely to undergo definitive repair if they recur.^7^

The use of prosthetic mesh in elective clean-contaminated or contaminated cases remains a controversial topic, despite randomized clinical trials and meta analyses demonstrating its safety and efficacy.^14,15,16,17^ There are considerably less high-quality data to guide decision-making in the emergent setting, and traditional dogma would lead many surgeons to avoid mesh or use biologic mesh.^18,19^ Some existing studies have found similar results to elective cases, finding prosthetic mesh use in emergent hernia repairs to have similar postoperative safety and reduced recurrence risk compared with no mesh.^2,20,21,22,23^ Others have found increased short-term risks in the emergent setting associated with specific types of mesh, such as biologic mesh, or mesh placement, such as the retromuscular space.^24,25^ However, these studies are limited to small, single institution samples or short-term outcomes.

We were able to analyze this issue at a population level. Our data demonstrated an association between any mesh use and the reduced risk of long-term recurrence. Additionally, the risk of mesh explantation was low, at approximately 3% across all open emergent cases. And perhaps most notably, mesh explantation rates were not significantly different for cases likely to have clean (no concurrent enterectomy) vs contaminated (concurrent enterectomy) wounds. These data build on available evidence evaluating the long-term safety and efficacy of mesh use in emergent hernia repair.^2,14,15,17,20,21,22,23^

We were able conduct a unique population-level analysis, with our data showing that mesh use was associated with a nearly one-third reduction in the risk of long-term recurrence. In evaluating safety, the risk of mesh explantation in our study was low at 3.2% at 10 years across all open emergent cases. There are limited data with which to compare this, but 1 study found mesh explantation rates for elective ventral hernia repairs to be 1.5% at 5 years.^26^ Most notably, mesh explantation rates were not significantly different for cases likely to have clean (no concurrent enterectomy) vs contaminated (concurrent enterectomy) wounds. These findings build on available evidence evaluating safety and efficacy of mesh use in emergent repair and suggest that many patients may not sacrifice safety for a more definitive up-front repair, even in nonideal circumstances.^2,14,15,17,20,21,22,23^

While evidence clearly shows lower postoperative morbidity for patients undergoing elective hernia repairs, literature evaluating recurrence rates for emergent vs elective repairs is mixed.^3,4^ For example, 2 studies demonstrated up to 4-fold higher recurrence in patients undergoing emergent repair.^3,27^ Alternatively, other studies have failed to show differences in recurrence based on case urgency, despite the clear differences in morbidity and mortality.^4,5,6^ These studies are limited by inconsistent risk adjustment, small cohorts, and short-term follow-up. Our population-level study found that the long-term, risk-adjusted reoperation rate for recurrence after emergent repair was similar to reported rates for elective repair.^7^ There are a few possible explanations for this. One is that previously reported increases in risk for recurrence after emergent hernia repair may be attributable to comorbidities, repair approach, and use of mesh; therefore, recurrence rates were similar when adjusting for these factors. Alternatively, patients undergoing initial emergent repair may have higher rates of clinical recurrence but be less likely to have their recurrences operatively repaired, potentially due to prohibitive comorbidities or lack of access.

This study has important implications for the management of emergent ventral hernias. For surgeons, this study suggests that the use of mesh, when possible, is associated with reduced operative hernia recurrence while maintaining a low likelihood of requiring mesh explantation, even in the setting of bowel resection. While emergent operations prioritize different factors, and mesh may not be suitable for every case, mesh was used in fewer than one-third of cases overall and less than one-fifth of cases with enterectomy. This suggests substantial underutilization of mesh in this setting. Our finding that long-term reoperation rates for recurrence for emergent repairs are comparable with rates reported for elective repairs challenges the traditional reluctance to pursue definitive repairs in the emergent setting.^7^ With definitive approaches, patients undergoing emergent open ventral hernia repair may achieve durable outcomes, avoiding subsequent operations or the adverse quality-of-life outcomes associated with nonoperatively managed recurrences.

### Limitations

This study has several important limitations. First, the retrospective nature of the study renders it susceptible to selection bias and precludes causation. Administrative claims data lack clinical details such as enterotomies, hernia size, the type of mesh used, the layer of the abdominal wall where the mesh was placed, and primary vs incisional-ventral hernias, which may impact mesh infection and recurrence rates.^28,29,30^ This limits our ability to assess what the best approach to mesh placement in this setting is (in terms of type and location), which has been the subject of other smaller studies.^17,25^ However, the population-level analysis of outcomes after emergent hernia repair among hernia specialists and emergency general surgeons, suggesting that even across a broad variety of hernia sizes and mesh type and placement approaches, there may be a benefit, and the need for enterectomy may not necessarily be a contraindication.

Second, the Medicare population may not be generalizable to all patients undergoing emergent hernia surgery. Reoperative recurrence rates may be lower than expected, given the older patient population with an increased comorbidity burden. We could not measure clinical recurrence that did not require reoperation, which can still interfere with quality of life for older adults. However, recurrences requiring a reoperation are likely the recurrences with the most significant morbidity. It is reasonable to assume, based on existing studies, that clinical recurrence follows the same pattern as operative recurrence and is lower in the setting of mesh.^3^ Studies measuring clinical recurrences are significantly limited in their sample size due to the need for patient-reported data.

Additionally, while we used mesh explantation as a surrogate for infection, we cannot identify mesh complications treated nonoperatively or distinguish between mesh excised for infection vs excision for other reasons, such as pain. However, existing literature indicates that infection is the most common reason for explantation, and we have no reason to believe that the rationale behind explantation would be markedly different between the cohorts with and without enterectomy.^31^

## Conclusions

This cross-sectional study found that mesh use in emergent hernia repairs was associated with a significantly lower reoperation rate for recurrence at 10 years. Mesh explantation rates were low, with no increased risk of explantation in the setting of a concurrent bowel resection. These data suggest that surgeons may consider more consistent use of mesh in the emergent setting, potentially offering better definitive long-term outcomes without compromising safety.

## Notes

Supplement 1. **eFigure 1.** Stepwise Patient Inclusion/Exclusion and Final Index Ventral Hernia Repair Cohort Study Size **eFigure 2**. Annual Incidence of Urgent and Emergent Inpatient Ventral Hernia Repairs From 2011-2021 **eMethods. **Race and Ethnicity Use in Medicare Claims Data **eTable 1.** *ICD-9/10* and *CPT* Codes Used to Identify Initial Ventral Hernia Repair Operation and Operative Hernia Recurrence **eTable 2. **Cumulative Hazard and Hazard Ratios by Exposure
eReferences

Supplement 2.
Data Sharing Statement
