# Supplementary material for: Long-Term Recurrence and the Safety of Mesh Use After Emergency Ventral Hernia Repair
Source: JAMA Netw Open. 2025 Nov 18;8(11):e2544303. doi: 10.1001/jamanetworkopen.2025.44303 (PMC12628106; doi:10.1001/jamanetworkopen.2025.44303)
Supplement: Supplement 1. — eFigure 1. Stepwise Patient Inclusion/Exclusion and Final Index Ventral Hernia Repair Cohort Study Size eFigure 2. Annual Incidence of Urgent and Emergent Inpatient Ventral Hernia Repairs From 2011-2021 eMethods. Race and Ethnicity Use in Medicare Claims Data eTable 1. ICD-9/10 and CPT Codes Used to Identify Initial Ventral Hernia Repair Operation and Operative Hernia Recurrence eTable 2. Cumulative Hazard and Hazard Ratios by Exposure eReferences [file jamanetwopen-e2544303-s001.pdf]

# Supplemental Online Content

Isenberg EE, Fry BT, Sinamo J, et al. Long-term recurrence and the safety of mesh use after emergency ventral hernia repair. *JAMA Netw Open*. 2025;8(11):e2544303.
doi:10.1001/jamanetworkopen.2025.44303

**eFigure 1.** Stepwise Patient Inclusion/Exclusion and Final Index Ventral Hernia Repair Cohort Study Size
**eFigure 2**. Annual Incidence of Urgent and Emergent Inpatient Ventral Hernia Repairs From 2011-2021
**eMethods.** Race and Ethnicity Use in Medicare Claims Data
**eTable 1.** *ICD-9/10* and *CPT* Codes Used to Identify Initial Ventral Hernia Repair Operation and Operative Hernia Recurrence
**eTable 2.** Cumulative Hazard and Hazard Ratios by Exposure
**eReferences**

This supplemental material has been provided by the authors to give readers additional information about their work.

© 2025 Isenberg EE et al. *JAMA Network Open*.

**eFigure 1.** Stepwise Patient Inclusion/Exclusion and Final Index Ventral Hernia Repair Cohort Study Size

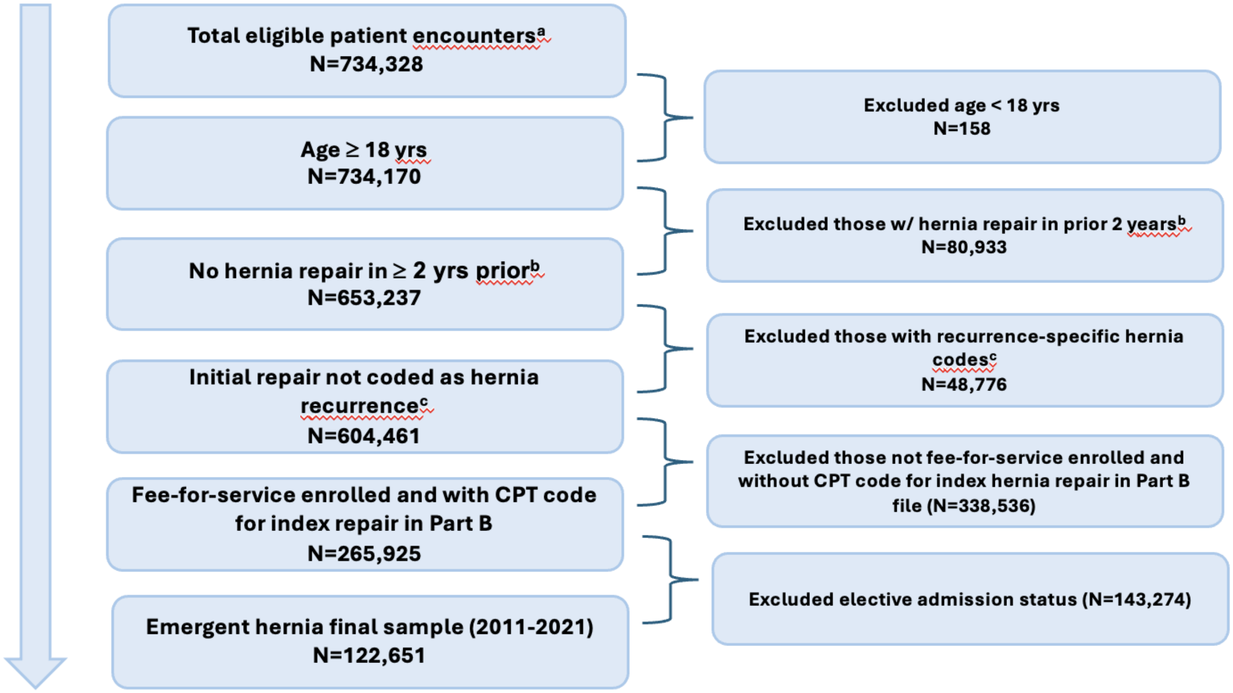


[a]Patients initially identified using International Classification of Diseases 9th and 10th Edition (ICD-9/10) procedure codes, which were cross referenced with the corresponding ICD-9/10 diagnosis codes (**eTable 1 in Supplement**)

[b]Because not all recurrent hernias repairs are coded as such, we reduced the possibility of mis-identifying index hernia repairs by excluding patients with an additional ventral hernia repair admission dating back at least 2 years prior to their year of index surgery.

[c]Recurrent hernia specific Current Procedural Terminology (CPT) codes were 49565, 49566, 49656, 49657

© 2025 Isenberg EE et al. *JAMA Network Open*.

**eFigure 2**. Annual Incidence of Urgent and Emergent Inpatient Ventral Hernia Repairs From 2011-2021.[a]

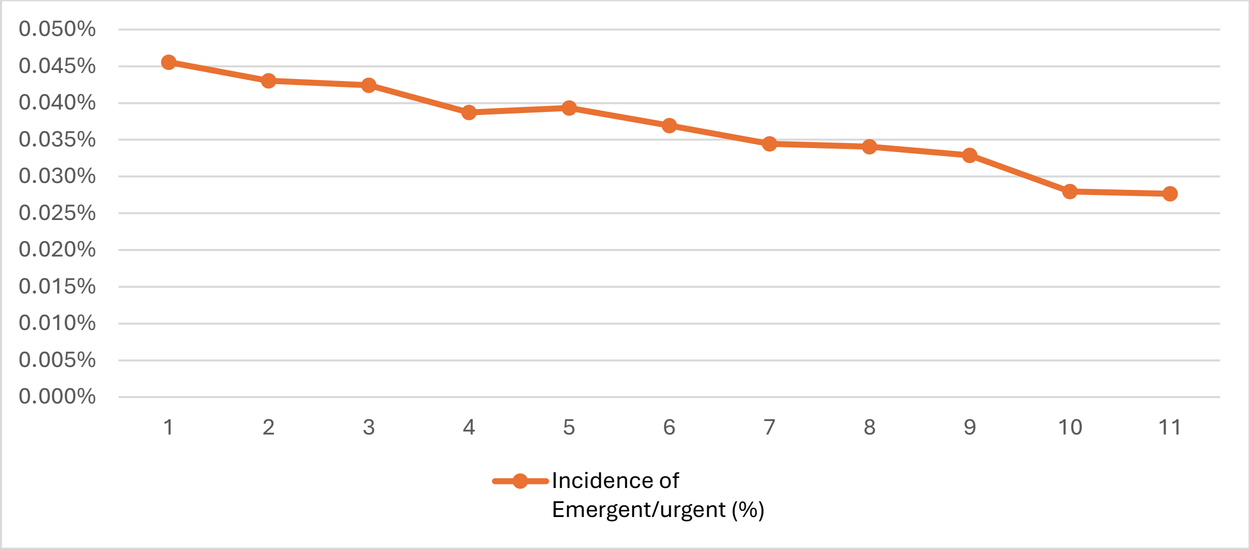


[a]Incidence reported as number of annual repairs divided by annual Medicare enrollees for a specific year

© 2025 Isenberg EE et al. *JAMA Network Open*.

**eMethods.** Race and Ethnicity Use in Medicare Claims Data

Patient race and ethnicity are included as identifiers in the Medicare claims database.[1] This data was collected by the Social Security Administration when individuals applied for Social Security. These identifiers were used to identify patient race and ethnicity in this study, and are represented in the following standard, fixed categories: Asian, Black, Hispanic, North American Native, White, other race/ethnicity, and unknown race/ethnicity. Only a single category could be selected during our study period, and no additional information is available in Medicare claims data regarding the composition of the "other race" category. Race and ethnicity were included as they have been found to be associated with the presentation, management, and outcomes of hernias and hernia repair.[2-4]

© 2025 Isenberg EE et al. *JAMA Network Open*.

**eTable 1.** *ICD-9/10* and *CPT* Codes Used to Identify Initial Ventral Hernia Repair Operation and Operative Hernia Recurrence[a]

| | |
|---|---|
| **Codes Used to Identify Ventral, Incisional, Umbilical, or Epigastric Hernia** | |
| ***ICD-9 Diagnosis Codes*** | 551.1, 551.2, 551.20, 551.21, 551.29, 552.1, 552.2, 552.20, 552.21, 552.29, 553.1, 553.2, 553.20, 553.21, 553.29 |
| ***ICD-9 Procedure Codes*** | 53.4, 53.41, 53.42, 53.43, 53.49, 53.5, 53.51, 53.59, 53.6, 53.61, 53.62, 53.63, 53.69 |
| ***ICD-10 Diagnosis Codes*** | K42, K42.0, K42.1, K42.9, K43, K43.0, K43.1, K43.2, K43.6, K43.7, K43.9 |
| ***ICD-10 Procedure Codes*** | 0WQF0ZZ, 0WMF0ZZ, 0WQF0ZZ, 0WQF3ZZ 0WQF4ZZ, 0WQFXZZ, 0WUF07Z, 0WUF0JZ, 0WUF0KZ, 0WUF47Z, 0WUF4JZ, 0WUF4KZ |
| ***CPT Codes*** | 49560, 49561, 49585, 49587, 49652, 49653, 49654, 49655, 49570, 49572 |
| **Additional Procedure Codes** | |
| ***Robotic-assisted Codes*** | *ICD-9*: 1741, 1742, 1743, 1744, 1749<br>*ICD-10*: 8E0W0CZ, 8E0W3CZ, 8E0W4CZ, 8E0W4CZ, 8E0W7CZ, 8E0W8CZ, 8E0WXCZ<br>*CPT*: S2900 |
| ***Component Separation*** | CPT code 15734 |
| ***Operative Hernia Recurrence*** | CPT codes 49565, 49566, 49656, 49657 |

ICD-9: International Classification of Diseases 9 Edition; ICD-10: International Classification of Diseases 10th Edition; CPT: Current Procedural Terminology.

© 2025 Isenberg EE et al. *JAMA Network Open*.

**eTable 2.** Cumulative Hazard and Hazard Ratios by Exposure

| Category | 10- year cumulative hazard (%) (95% CI) | Overall hazard ratio (95% CI) | p-value |
|---|---|---|---|
| Reoperative recurrence rate for umbilical versus incisional/ventral (Figure 1b) | | | |
| Umbilical | 10.98 (10.57-11.39) | 0.54 (0.52-0.57) | <0.001 |
| Incisional/Ventral | 19.21 (18.76-19.65) | | |
| Reoperative recurrence rate for mesh versus no mesh repairs (Figure 2) | | | |
| Mesh | 12.96 (12.48-13.44) | 0.66 (0.63-0.69) | p<0.001 |
| No mesh | 18.86 (18.39-19.33) | | |
| Mesh explantation for enterectomy versus no enterectomy (Figure 3) | | | |
| Enterectomy | 3.77 (2.77-4.77) | 1.20 (0.91-1.59) | p>0.05 |
| No enterectomy | 3.16 (2.89-3.42) | | |

© 2025 Isenberg EE et al. *JAMA Network Open*.

## eReferences


1. Race/ethnicity (from Medicare EDB) | ResDAC. Resdac.org. Published 2022. Accessed January 13, 2022. https://resdac.org/cms-data/variables/raceethnicity-medicare-edb.
2. Bowman K, Telem DA, Hernandez-Rosa J, Stein N, Williams R, Divino CM. Impact of race and socioeconomic status on presentation and management of ventral hernias. *Arch Surg.* 2010;145(8):776-780.
3. Vu JV, Gunaseelan V, Dimick JB, Englesbe MJ, Campbell DA, Jr., Telem DA. Mechanisms of age and race differences in receiving minimally invasive inguinal hernia repair. *Surg Endosc.* 2019;33(12):4032-4037.
4. Asolati M, Huerta S, Sarosi G, Harmon R, Bell C, Anthony T. Predictors of recurrence in veteran patients with umbilical hernia: single center experience. *Am J Surg.* 2006;192(5):627-630.


© 2025 Isenberg EE et al. *JAMA Network Open*.
