# Supplementary material for: Long-Term Recurrence and the Safety of Mesh Use After Emergency Ventral Hernia Repair
Source: JAMA Netw Open. 2025 Nov 18;8(11):e2544303. doi: 10.1001/jamanetworkopen.2025.44303 (PMC12628106; doi:10.1001/jamanetworkopen.2025.44303)
Supplement: Supplement 2. — Data Sharing Statement [file jamanetwopen-e2544303-s002.pdf]

Data Sharing Statement

Data

Data available: No

Additional Information

Explanation for why data not available: CMS data sharing agreements
